# The Behavior of VLF/LF Variations Associated with Geomagnetic Activity, Earthquakes, and the Quiet Condition Using a Neural Network Approach

**DOI:** 10.3390/e20090691

**Published:** 2018-09-11

**Authors:** Irina Popova, Alexandr Rozhnoi, Maria Solovieva, Danila Chebrov, Masashi Hayakawa

**Affiliations:** 1 Institute of Physics of the Earth RAS, Bolshaya Gruzinskay, 10-1, Moscow 123242, Russia; 2 Kamchatska Branch of Geophysical Survey of RAS, Boulevard Piypa, Petropavlovsk-Kamchatsky 683006, Russia; 3 Advanced Wireless Communications Research Center, University of Electro-Communications, Chofu, Tokyo 182-8585, Japan

**Keywords:** earthquake precursors, magnetic storm, neural network, low frequency electromagnetic signals

## Abstract

The neural network approach is proposed for studying very-low- and low-frequency (VLF and LF) subionospheric radio wave variations in the time vicinities of magnetic storms and earthquakes, with the purpose of recognizing anomalies of different types. We also examined the days with quiet geomagnetic conditions in the absence of seismic activity, in order to distinguish between the disturbed signals and the quiet ones. To this end, we trained the neural network (NN) on the examples of the representative database. The database included both the VLF/LF data that was measured during four-year monitoring at the station in Petropavlovsk-Kamchatsky, and the parameters of seismicity in the Kuril-Kamchatka and Japan regions. It was shown that the neural network can distinguish between the disturbed and undisturbed signals. Furthermore, the prognostic behavior of the VLF/LF variations indicative of magnetic and seismic activity has a different appearance in the time vicinity of the earthquakes and magnetic storms.

## 1. Introduction

Very-low-frequency/low-frequency (VLF/LF) signal monitoring (range, 10–50 kHz) has become one of the main research methods in the studies on the state of the lower ionosphere and the upper atmosphere. The application of this method for analyzing signal variations that are associated with seismic activity was applied in [1,2,3,4]. In addition to the conventional night-time fluctuation method, the natural time analysis to the subionospheric VLF data has been presented in [5]. It was shown that the lower ionosphere, as seen by VLF propagation, exhibited critical characteristics from two weeks up to two days before the main shock of the disastrous 2016 Kumamoto earthquakes (EQ) (15 April 2016).

The main problem in applying this method for earthquake prediction is distinguishing the signal anomalies of seismic origin against the background of global perturbations, which are caused in the lower ionosphere by magnetic storms and sub-storms, and proton and electron fluxes. It was previously established that magnetic storms and solar energetic particle fluxes can induce phase and amplitude variations in the VLF signal [6,7,8,9]. The correlation of the phase and amplitude variations in the LF signal to the disturbance storm time (DST) index, as well as the correlation of the outer-zone particles (protons and electrons) to the high-pitch angle was revealed in [10,11,12]. In this work, we applied a neural network method for estimating the VLF/LF signal sensitivity to geomagnetic and seismic activity.

It should be noted, however, that other methods based on entropy have been recently found that when analyzing the earthquake time series in a new time domain termed natural time [13], it is observed that the entropy plays an important role. Specifically, upon analyzing the seismic time series in the Chiapas region before the occurrence of the M8.2 earthquake on 7 September 2017 in Mexico, the entropy change under time reversal exhibits a minimum almost three months before, and in particular on 14 June 2017 [14]. The study of the complexity measures associated with the fluctuations of either the entropy defined in natural time and/or the entropy change under the time reversal of the seismicity in the Chiapas region from 1 January 2012 until 20 October 2017, was continued in [15].

The neural network approach was developed to understand how the brain solves the problem of learning, pattern recognition, and decision making. The ability of the neural network to approximate any continuous function with arbitrary accuracy was used in a variety of geophysical applications. The neural network technology was applied in the tasks of parameter estimation, filtering, classification, and prediction. The supervised neural networks were used for function approximation and inversion problems in Refs. [16,17,18,19]. The recurrent neural network was constructed for predicting the magnetic storm intensity [20]. Forecasting the earthquake magnitudes based on the neural networks were considered in [21,22]. A new signal processing algorithm (WANEH) inspired by the deep learning paradigm was presented in [23]. It combines wavelets, neural networks, and a Hilbert transform. The probabilistic receiver operating characteristics (ROC) method [24], supported by the confidence ellipses [25], was employed for determining the anomaly detection threshold and the statistical significance of the detector’s outcome. The authors proposed a unique Deep Neural Network structure for reconstructing the normal behavior and the time–frequency analysis of the residual signal in relation to the probabilistic ROC. The method was shown to be able to automatically detect anomalies in the seismic electrical signal, which could be used to predict earthquake activity. Furthermore, the method can be used in combination with crowdsourcing of smartphone data to locate road defects. In the road anomaly case, the proposed algorithm was compared to two known road anomaly detection methods based on a probabilistic method [26], and based on wavelet decomposition and support vector machines [27]. The comparison confirms the good performance of the WANEH algorithm. 

The artificial neural network was applied to forecast gasoline consumption in [28]. The neural network based on the backpropagation algorithm was implemented using the cross entropy error function in the training stage. The cross entropy function was proven to accelerate the back-propagation algorithm, and to provide good overall network performance with relatively short stagnation periods. A method for estimating the VLF/LF signal sensitivity to seismic processes using a neural network approach based on the backpropagation technique was proposed in [29,30]. The trained neural network was applied in the forecasting mode for the automatic detection of anomalous changes in the VLF/LF signal related to the seismic activity above a certain threshold.

For further study, we develop a neural network method to find the geomagnetic VLF/LF signal anomalies. We compare the behavior of the seismic and geomagnetic VLF/LF signal anomalies in the time vicinity of the earthquakes and magnetic storms. We also test the neural network on the set of time intervals with quiet geomagnetic and seismic conditions for analyzing the behavior of VLF/LF signal in many aspects.

## 2. Materials and Methods

**Figure 1:**
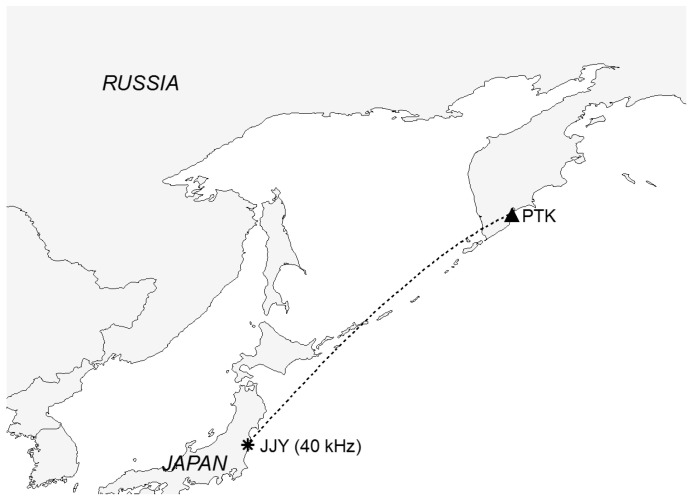
A map showing the positions of the receiver in Petropavlovsk-Kamchatsky (PTK) and the transmitter JJY (40 kHz).

The database for our study is based on the VLF/LF signals measured during monitoring in the Kuril Kamchatka region. We analyzed the observations of 2004–2007. The receiver was installed in Petropavlovsk-Kamchatski, and the radio transmitter JJY was located in Japan (see Figure 1).

The database included the amplitudes and phases of the VLF/LF signals and the corresponding parameters of regional seismicity (the magnitudes of seismic events *M* and distances *D* from the earthquake epicenters to the axis of the receiver–transmitter path, hypocentral depth *H*, ratio *R*/*D*, between the radius *R* of the area of precursory manifestations and distance *D*). These parameters were taken from the earthquake catalog (https://earthquake.usgs.gov/contactus/golden/neic.php). We also used information about the global perturbations of the lower ionosphere caused by the magnetic storm and relativistic high energy electron flux (https://www.swpc.noaa.gov/).

**Figure 2:**
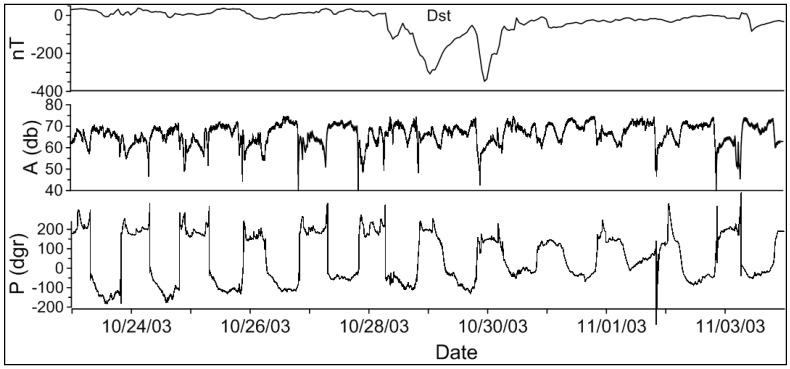
The anomalies in the amplitude and phase of the JJY signal during a super strong magnetic storm ‘Halloween” over 28–31 October 2003. The top panel shows the disturbance storm time (DST) index of magnetic activity, the two next panels show the amplitude and phase of the JJY signal.

**Figure 3:**
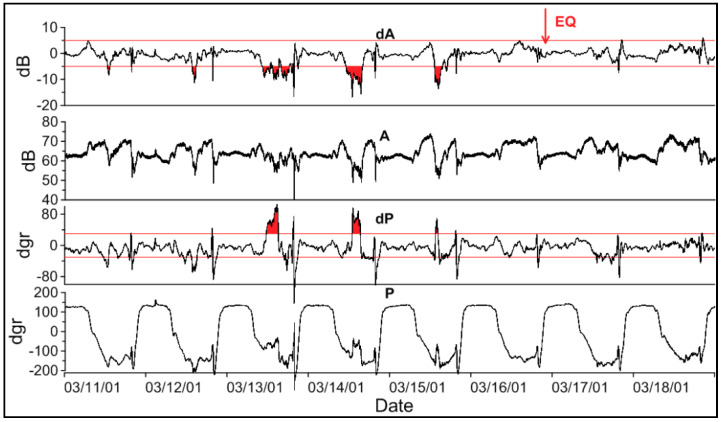
The anomalies in the low-frequency (LF) signal observed in Petropavlovsk-Kamchatski before the earthquake occurred on 17 March 2001 (*M* = 5.5). Top-down: the residual amplitude, the amplitude of the JJY signal, the residual phase, and the phase of JJY signal. The arrow shows the occurrence time of the earthquake. The red lines are the level of two standard deviations. The filled areas highlight the anomalies in the amplitude and phase of the LF signal related to the earthquake.

The examples of the anomalies in the amplitude and phase of the real LF data measurements at the Petropavlovsk-Kamchatski station during a super strong magnetic storm in 28–31 October 2003 (DST ~ −400 nT), and in connection with the earthquake on 17 March 2001 (*M* = 5.5) are shown in Figure 2 and Figure 3 correspondingly. In Figure 3, besides the real data we showed the differences or residual signals. For each month, an average background (model) signal was calculated based on data from the ‘quiet days’, and then the residual signals of amplitude and phase (dA and dP) were calculated as the difference between the observed and model signals.

The anomalies in the amplitude began together with the sudden commencement and continued during the main and recovery stages of the storm. The phase anomalies were observed during the main and recovery stages.

In Figure 3 besides the real data, we show the differences or residual signals. For each month, an average background (model) signal is calculated based on data from the ‘quiet days’, and then the residual signals of amplitude and phase (dA and dP) are calculated as the difference between the observed and model signals. The anomalies of the signal in this case were observed 3–4 days before the earthquake.

In order to estimate the behavior of the VLF/LF variations associated with the geomagnetic activity, earthquakes, and quiet conditions, we applied the neural network technique for the prediction and classification problem. We also used supervised learning because we knew both the input and corresponding output values for creating the training dataset. At the first step, the neural network was taught the relationship between the input and output from this training set. At the second step, the recognition (prediction) procedure was executed on the previously trained neural network for the unknown data that we want to recognize.

**Figure 4:**
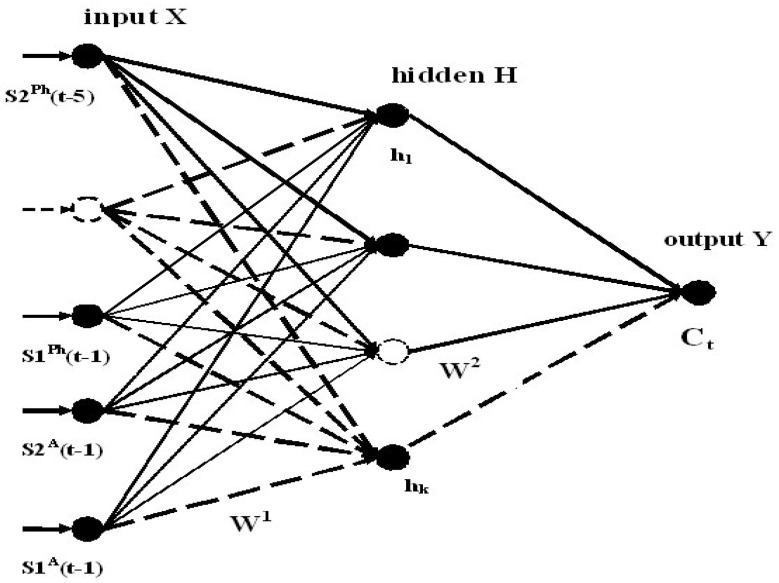
Three-layer perceptron of the backpropagation neural network used for recognition (prediction) of disturbed and quiet signals. The input signal is composed of the means and variances values of the phases (S1^Ph^(t-n) and S2^Ph^(t-n), respectively), and amplitudes (S1^A^(t-n) and S2^A^(t-n), respectively) during the night time for five days before the prognostic day (n varies from 1 to 5). The corresponding level of the correlation C_t_ is used as the output data.

The most common example of the supervised learning is the multi-layer perceptron. We used the three-layer perceptron, as illustrated in Figure 4. The first and third layers were referred to as the input and output layers, respectively. The second layer was referred to as the hidden layer. The neurons of each layer were shown by circles. The neurons of the previous layer were connected with the neurons of the next layer. The lines between the layers represented the weights that are applied to the outputs of each layer. The backpropagation technique [31], based on the multi-layer perceptron, is described by the following formula:

(1) $$y_{i}^{l}=f(\sum_{j}W_{ij}^{l}x_{j}),\;$$

where $y_{i}^{l}$ is the output signal of the neuron i of the layer l, $W_{ij}^{l}$ are the weight connections between the neurons of layers l − 1 and l, $x_{j}$ is the value of the neuron j-th of layer l − 1, and *f* is the neuron response function. In our case, the neuron response function is defined by the logistic function:

(2) $$f(z)=\frac{1}{1+e^{-z}}\;$$

Thus, the input signal propagated forward from one layer to another layer.

At the training stage, our purpose was to obtain the output signal $y_{i}$ on the third layer that minimizes the total standard error:

(3) $$E=\sum_{p}\sum_{i}{\left(y_{i}-y_{i}^{t}\right)}^{2}.$$

The summation was carried out for each training example p over all neurons i of the output layer. The “target” value $y_{i}^{t}$ represented the sample value for the corresponding training example. The real value $y_{i}$ represented the value of the output neuron that was formed as a result of signal propagation (1). The values of the weights were determined by back-propagating the errors between the inputs and outputs for minimizing error (3) by the gradient descent technique:

(4) $$\Delta W_{ij}^{\left(n\right)}=-\alpha \frac{\partial E}{W_{ij}}+\beta \Delta W_{ij}^{\left(n-1\right)},$$

where $\Delta W_{ij}^{\left(n\right)}$ is the increment of the weight connection at the step n, $\Delta W_{ij}^{\left(n-1\right)}$ is its increment at the previous step, α and β are the internal parameters of the neural network.

The recognition procedure is carried out at one passage of the recognizable signal from the input to the output, and it uses the weight connections that are specified in the teaching process.

We applied the neural approach for the recognition (prediction) the disturbed and undisturbed VLF/LF signals. The optimal teaching database was formed after many experiments on teaching and testing the neural networks. As a result, we selected 80 examples for the neural network teaching. Each example included the input and output data. The input vector X consisted of the mean values and variances that were calculated from the amplitudes and phases of the signals during the night time of five days before the prognostic day. Thus, the number of neurons in the input layer was 20. In the process of NN teaching, we should establish the correspondence between the input and output. The output vector Y was equal to 1 for 40 examples of the disturbed VLF/LF signals. The output vector Y was equal to 0 for 40 examples of the quiet signals in the absence of seismic and geomagnetic activity. The number of neurons in the hidden layer was 8. The NN architecture is illustrated in Figure 4.

The mean and variances values of the amplitude and phase are denoted by S1^A^, S2^A^, and as S1^Ph^ and S2^Ph^ respectively. In the output layer of the NN, a single neuron has the sense of the correlation coefficient (by the absolute value) C_t_.

The result of the recognition is formed as the output after teaching the neural network. In our case, the final result represented by the value of the coefficient of correlation associated with the quiet or disturbed signals on the sixth day, because at the stage of teaching, we set the correspondence between the characteristic changes in the VLF/LF signals five days before a prognostic day and the correlation coefficient on the sixth day. The correlation coefficient varied from 0 to 1 due to the interpolation properties of the neural network. We assumed that when the correlation was above 0.5, the classifying the event into the disturbed signal was probable. We interpreted the value of the output as the characteristic classifying the events into the disturbed and quiet signal. In this case, the neural network was used not only for the prediction, but also for the signal classification.

## 3. Results

This study addresses the analysis of the behavior of the VLF/LF signals caused by the geomagnetic activity. Another objective of the study was to distinguish between the anomalies of non-seismic and seismic origin. For the comparison, we should also understand the behavior of the VLF/LF signals corresponding to the quiet geomagnetic and seismic conditions. For this aim, we should examine the variations in the phase and amplitude of VLF/LF signals on the set of the time intervals including the day of the magnetic storms and seismic events. We should also analyze the behavior of the signal during the quiet days.

For this purpose, we moved the previously trained neural network along the time axis. We formed the input vector using the five days before the prognostic day of the selected time interval. We applied this vector on the input layer of the trained neural network and received the value of the coefficient of correlation on the sixth day in the output layer. After this, we repeated the recognition procedure on the same NN with a shift by one day, etc. Thus, we moved the 5-day time window of the input vector along the given time interval, with a one-day shift. As a result, we obtained the values of the correlation for the entire time interval, including the days of the geomagnetic or seismic activity, and the quiet days.

**Figure 5:**
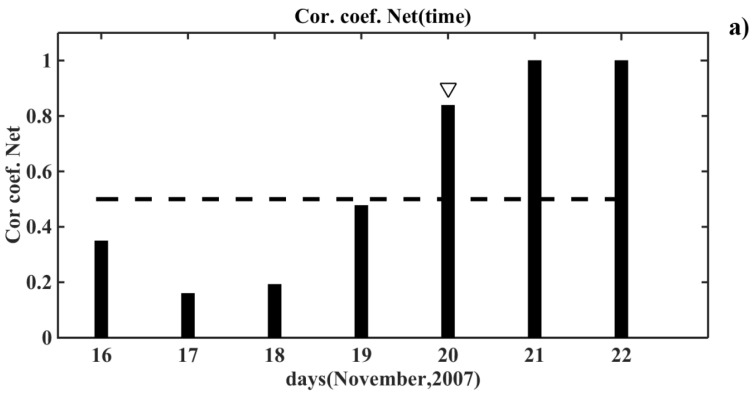

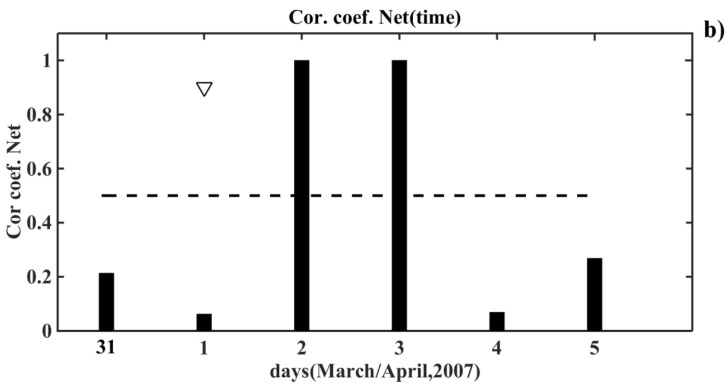
The neural network detection of the anomalous changes caused in the very-low-frequency/low-frequency (VLF/LF) signal by the magnetic storms (the magnetic activity index DST ≤ −60 nT): (**a**) in the time interval of 16–22 November 2007 (the day of magnetic storm was 20 November); (**b**) in the time interval of 31 March to 5 April 2007 (the day of magnetic storm was 1 March). Each column in Figure 5a,b represents the values of the coefficient of correlation on a certain day of the considered time period. The dashed line in this diagram represents the threshold value of the coefficient of correlation, which is equal to 0.5. The arrow marks the first day of the magnetic storm.

The trained neural network was used for detecting the anomalous changes in the VLF/LF signal caused by the magnetic activity with the magnetic activity index DST ≤ −60 nT. Besides, the considered time intervals did not include the time vicinity of the day (including the day itself) when the flux of relativistic electrons exceeded the given threshold. We also excluded from the consideration the time intervals with the day of the seismic event with the magnitude *M* ≥ 5.5. The examples of this analysis are illustrated in Figure 5a,b, which shows the correlation coefficients on the considered time intervals obtained as the result of NN prediction.

The dashed line in the graphs depicts the level of the correlation coefficient equals to 0.5. The arrow marks the first day of the magnetic storm. We can see that the magnetic storm-induced disturbances in the signals arose on the very day of the magnetic activation (Figure 5a), and in addition, a few days after it or after the day of the onset of magnetic activation (Figure 5b). Indeed, the disturbance in the signal occurred not only on the day of the magnetic storm, but also the day after it [10,11,12]. For example, if a magnetic storm began in the daytime, then as a rule, the disturbance in the signal occurred only the next day.

We analyzed five time intervals with the days of the magnetic storms overall. These days are presented in Table 1 with information about the magnetic activity index DST.

We did not detect the disturbances in the signals before the days of the magnetic storms in all considered cases. In one case of the five, the disturbances in the signals caused by magnetic storms were not detected absolutely. These results corresponded to the conclusions that had been obtained in previous work [10,11,12].

For comparing the behavior of VLF/LF signals that were subject to the geomagnetic and seismic activity, we should be reminded of the results concerning the prediction of seismic events with magnitudes *M* ≥ 5.5 [29]. In a previous study, we tested 12 time intervals with a length of 6–8 days of 2004, 2005, 2006, and 2007. Each time interval contained the day of the seismic event with magnitudes *M* ≥ 5.5.

**Figure 6:**
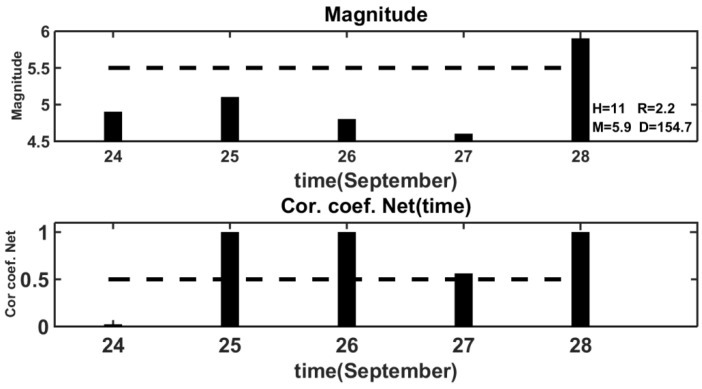
The neural network prediction of the seismic event for 24–28 September 2006. Each column in the upper diagram in Figure 4 represents the value of magnitude of the earthquakes which occurred on a certain day of the considered time period. When the magnitude is equal to or more than 5.5, the corresponding column is marked with the following parameters: magnitude *M*, depth *H*, distance *D* from the epicenter of the earthquake to the axis of the “transmitter–receiver” line, and the ratio *R*/*D*. The dashed line depicts the threshold at which *M* ≥ 5.5. The corresponding values of the coefficient of correlation are shown in the lower diagram. The dashed line here represents the threshold value of the coefficient of correlation of 0.5.

The example of NN predicting the behavior of the VLF/LF signals caused by the earthquakes with *M* ≥ 5.5 is illustrated in Figure 6.

We considered the results of the NN prediction for the time interval from 24–28 September 2006 which included the day of the seismic event of 28 September 2006. In the first bar graph in Figure 4, the magnitudes for each day of the selected time interval are represented by the columns. The dashed line denotes the threshold value of the magnitude *M* = 5.5. The following parameters of the seismic event are indicated near the column denoting the seismic event with magnitude *M* ≥ 5.5: the magnitude *M*, the depth *H*, the distance from the epicenter to the receiver–transmitter axis *D*, and the *R*/*D* ratio. The values of the coefficient of correlation for the same days obtained as the result of NN prediction are shown in the second bar graph. These values were formed by the NN output, which was moved along the given time interval with a one-day shift. The dashed line in this diagram marked the coefficient of correlation as being equal to 0.5. The diagram in the bottom panel of Figure 4 shows that the correlation coefficients were higher than the threshold value of 0.5, three days before the earthquake of 28 September, and on the very day of the earthquake.

Thus, the NN recognized the earthquake-related disturbances in the signal on nine of the 12 time intervals considered. The information about the earthquakes predicted by the neural network is presented in Table 2.

An important fact is that the disturbances in the signal were not only predicted on the day of the earthquake but also before the earthquake for all nine seismic events [29,30], in contrast to the appearance of such disturbances in the signals on the very day of the magnetic activation and a few days after it, or after the day of the onset of magnetic activation, but not before.

The results of this comparison showed that the neural network analysis allowed the anomalies of the VLF/LF signals of seismic and magnetic origin to be distinguished in the time vicinities of the earthquakes and magnetic storms.

However, for making the final conclusion we need to examine the trained neural network on the set of the time intervals which do not include the days of seismic events with magnitudes *M* ≥ 5.5. Besides, the considered time intervals did not include the days when the magnetic activity index DST and the flux of relativistic electrons exceeded the given thresholds. We tested 89 days overall on different time intervals in 2004, 2005, 2006, and 2007.

**Figure 7:**
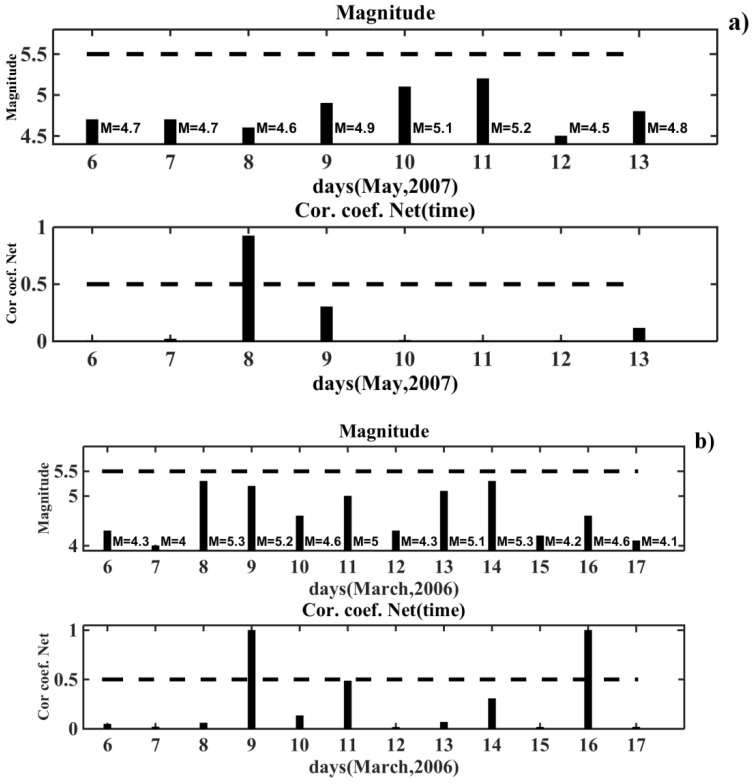
The neural network prediction on the time intervals with quiet seismic and geomagnetic conditions: (**a**) the results of the prediction for 6–13 May 2007; (**b**) the results of the prediction for 6–17 March 2006.

The results of NN recognition (prediction) of the behavior of the VLF/LF signals during the quiet seismic and geomagnetic conditions are illustrated in Figure 7a,b.

The same previously-trained neural network was used for the earthquake prediction. As an example, we considered the results of the NN prediction on the time interval from 6–13 May 2007 shown in Figure 7a. The columns and dashed lines in the top and bottom diagrams of Figure 7 have the same meaning, as in Figure 6. In the upper diagram, the magnitudes for each day of the selected time interval are indicated near the columns. The dashed line denotes the threshold value of the magnitude *M* = 5.5. We saw that the behavior of the coefficient of correlation on 8 May demonstrated a disturbance in the signal. It could be classified as a “false alarm”. Similar results were shown in the time interval from 6–17 March 2006 in Figure 7b. These “events” could be related to the unaccounted influence of the meteorological parameters of the atmosphere.

The “false alarms” were recognized by the neural network in 12 cases out of 89. Such “events” were sparse in the sense that a few days before and few days after them, there were no other “events”. These local “events” differed from the recognized seismic events, which were preceded by the disturbance of the signal over several days before the earthquake, and on the day of the earthquake. The results of this study show that the neural network can distinguish in time the signals disturbed by seismic activity from the quiet ones.

## 4. Discussion

The trained neural network detected the anomalous changes in the VLF/LF signal caused by the magnetic storms. The changes in the VLF/LF signal, indicative of magnetic activity, were identified in four of the five time intervals. The results of this study show that the pattern of the changes induced in the VLF/LF signals by the earthquakes and magnetic activity is different in time. In contrast to the behavior pattern of the magnetic activity in time, the neural network can predict the seismic events using the night-time anomalies in the VLF/LF signals as the precursors of the earthquakes.

Besides, the neural network can distinguish the signals disturbed by the seismic activity from the quiet ones. The detection of the local “false alarms” on the “quiet” time intervals is different from the detection of the seismic events, which are characterized by the disturbance of the signal several days before the earthquake, and on the day of the earthquake.

It is worth noting that we did not take into account the proton and electron fluxes, and the meteorological characteristics of the atmosphere. The combination of the disturbances caused in the signals by these factors, in addition to the magnetic activity, can complicate the pattern of the changes in the signals in time.

## Figures and Tables

**Table 1:** The five magnetic storms with magnetic activity index DST ≤ −60 nT for the considered five time intervals in 2004, 2005, and 2007.

N	Year	Month	Day	DST
**1**	2007	11	20	−63
**2**	2007	5	23	−63
**3**	2007	4	1	−63
**4**	2005	6	23	−66
**5**	2004	7	17	−80

**Table 2:** The nine seismic events with *M* ≥ 5.5 predicted by the neural network.

N	Year	Month	Day	Time	Latitude (°)	Longitude (°)	Depth (km)	*M*	*D* (km)	*R*/*D*
**1**	2006	9	28	1:36	46.5	153.3	11	5.9	154.7	2.22
**2**	2004	9	13	3:0	44	151.4	8	6.1	223.7	1.87
**3**	2004	11	2	13:4	38.8	142.8	23	5.6	26.1	9.8
**4**	2007	1	11	20:34	43.5	147.1	10	5.5	45.7	5.07
**5**	2005	3	11	18:47	43.1	144.7	54	5.5	187.7	1.23
**6**	2005	3	16	13:23	43.5	146.9	39	5.6	59.4	4.3
**7**	2005	8	1	4:40	46.9	153.9	16	5.7	156.8	1.8
**8**	2004	5	29	3:47	37.7	141.9	29	5.8	51.9	5.9
**9**	2004	7	21	0:11	40.9	143.1	30	5.5	127.9	1.81
